# Molecular Characterization and Antibiotic Susceptibility of Non-PCV13 Pneumococcal Serotypes among Vaccinated Children in Cape Coast, Ghana

**DOI:** 10.3390/microorganisms10102054

**Published:** 2022-10-18

**Authors:** Richael O. Mills, Mohammed R. Abdullah, Samuel A. Akwetey, Dorcas C. Sappor, Johan A. Bolivar, Gustavo Gámez, Mark P. G. van der Linden, Sven Hammerschmidt

**Affiliations:** 1 Department of Molecular Genetics and Infection Biology, Interfaculty Institute for Genetics and Functional Genomics, Center for Functional Genomics of Microbes, University of Greifswald, 17489 Greifswald, Germany; 2 Department of Biomedical Sciences, School of Allied Health Science, University of Cape Coast, Cape Coast PMB TF0494, Ghana; 3 Department of Clinical Microbiology, School of Medicine, University of Development Studies, Tamale PMB TF0494, Ghana; 4 Department of Medical Laboratory Sciences, School of Allied Health Sciences, University of Cape Coast, Cape Coast PMB TF0494, Ghana; 5 Genetics, Regeneration and Cancer (GRC), University Research Centre (SIU), University of Antioquia (UdeA), Medellín 050010, Colombia; 6 Basic and Applied Microbiology (MICROBA), School of Microbiology, University of Antioquia Medellin, Antioquia 050010, Colombia; 7 German National Reference Center for Streptococci, Department of Medical Microbiology, University Hospital RWTH Aachen, 52074 Aachen, Germany

**Keywords:** *Streptococcus pneumoniae* 1, non-PCV13 serotypes 2, virulence genes 3, antibiotic susceptibility 4, MLST 5

## Abstract

Preventive strategies involving the use of pneumococcal conjugate vaccines (PCVs) are known to drastically reduce pneumococcal disease. However, PCV vaccination has been plagued with serotype replacement by non-PCV serotypes. In this study, we describe the prevalence and molecular characteristics of non-PCV13 serotypes (non-vaccine serotypes, NVTs) from pneumococcal carriage isolates obtained from children < 5 years old in Cape Coast, Ghana, after PCV introduction. The isolates were subjected to antibiotic susceptibility testing and multilocus sequence typing (MLST), and molecular techniques were used to detect the presence of virulence genes. Serotypes 11A, 13, 15B, 23B, and 34 formed the top five of the 93 NVT isolates. As such, 20 (21.5%), 49 (48.4%), and 70 (74.3%) isolates were non-susceptible to penicillin, tetracycline, and cotrimoxazole, respectively. Sixteen (17.2%) multidrug-resistant isolates were identified. However, non-susceptibility to ceftriaxone and erythromycin was low and all isolates were fully susceptible to levofloxacin, linezolid, and vancomycin. Whereas *pcpA*, *pavB*, *lytA*, and *psrP* genes were detected in nearly all serotypes, pilus islet genes were limited to serotypes 11A, 13, and 23B. MLST for predominant serotype 23B isolates revealed three known and seven novel sequence types (STs). ST172 and novel ST15111 were the most dominant and both STs were related to PMEN clone Columbia^23F^-26 (ST338). In conclusion, non-PCV13 serotype 23B was the most prevalent, with characteristics of rapid clonal expansion of ST172 and ST15111, which are related to international clones of the pneumococcus. Continuous monitoring of NVTs in Ghana is, therefore, essential, as they have the potential to cause invasive disease, show high antibiotic resistance, and attenuate the effects of PCV vaccination.

## 1. Introduction

Invasive diseases, such as meningitis and bacteremia, caused by *Streptococcus pneumoniae*, are a leading cause of mortality in children under five years, predominantly in low- and middle-income countries (LMIC). The introduction of pneumococcal conjugate vaccines (PCVs) is estimated to have reduced pneumococcal deaths by 51% by the end of 2015 [1]. Both PCV10 and PCV13 are included in vaccination schedules in childhood immunization programs in several countries. Post-PCV vaccination data have shown a drastic decline in invasive pneumococcal disease globally [2,3]. Although these PCVs have offered protection against vaccine serotypes (VTs), they have contributed to the insurgence of non-PCV serotypes. The increase in non-PCV serotypes is of concern, especially when these serotypes show resistance to antibiotics commonly used in treating invasive pneumococcal disease (IPD) [4,5]. Hence, the quest to develop higher-valency PCVs has been initiated. Interestingly, in 2021, the Food and Drugs Administration (FDA) in the United States of America approved the use of PCV20 (PREVNAR20) and PCV15 (VAXNEUVANCE) in adults 18 years old and above [6,7]. Subsequently, the European Medicines Agency (EMA) also approved the marketing of PCV20 (APEXXNAR) and PCV15 (VAXNEUVANCE) in Europe [8,9].

However, the phenomenon of serotype replacement by NVT is likely to continue, even with the future introduction of these higher-valency PCVs. Therefore, alternatives to PCVs have been proposed, including pneumococcal protein-based vaccine candidates (PBVCs). The proteins in these PBVCs must be conserved in all pneumococci in order to offer protection across all serotypes. Some PBVCs that have been proposed and are currently at different stages of clinical trials include pneumococcal choline-binding protein A (PcpA), pneumolysin (Ply), autolysin A (LytA), pneumococcal serine-rich repeat protein (PsrP), and pilus type 1 [10]. Common non-PCV serotypes that have been reported globally include serotypes 11A, 13, 15A, 15B,19B, 23A, 23B, and 34 [4,11,12]. In addition, some of these NVTs have been implicated in IPD [13,14].

With support from Gavi, The Vaccine Alliance, LMICs, including Ghana, have introduced PCV13 as part of their childhood immunization program. In 2012, Ghana introduced PCV13 vaccination among children following the 3+0 vaccine dosing schedule. However, there are concerns about the 3+0 vaccine dosing schedule, as many countries that implemented this schedule have reported increased NVTs and high residual carriage of VTs [15] compared to high-income countries that implemented the 2+1 vaccine dosing schedule [16,17,18].

Different NVTs have been reported in invasive disease based on geographical locations. In addition, there are indications of expansion of lineages of these NVTs causing invasive disease [13,19]. Assessing the dynamics of NVTs, their contribution to invasive disease as well as their clonal expansion is important to understand the overall impact of PCV vaccination. Such molecular epidemiological data on NVTs are limited in Ghana, although some post-vaccination carriage studies have been performed [20]. However, these studies were performed in the capital city of Ghana. In this study, we describe the antibiotic susceptibility patterns and molecular characteristics, including the detection of virulence genes and genetic relatedness of pneumococcal NVT obtained from children <5 years of age in Cape Coast, Ghana.

## 2. Materials and Methods

### 2.1. Pneumococcal Isolates and Serotyping

In February 2018, a total of 513 children under five years was enrolled into the pneumococcal carriage study. We selected children from kindergartens and childhood immunization centers in Cape Coast, Central region, Ghana. The isolation and detection of pneumococci were performed as described [11,21]. Briefly, nasopharyngeal swabs were cultured on sheep blood agar supplemented with 5 µg/mL gentamicin. Presumptive pneumococci were identified by optochin and bile solubility tests.

### 2.2. Antibiotic Susceptibility Testing

Susceptibility testing was determined using the disc diffusion method in accordance with the Clinical and Laboratory Standards Institute (CLSI) guidelines [22]. The isolates were tested for susceptibility to levofloxacin (5 μg), vancomycin (30 μg), linezolid (30 μg), clindamycin (2 μg), erythromycin (15 μg), tetracycline (30 μg), chloramphenicol (30 μg), cotrimoxazole (25 μg), and ceftriaxone (30 μg). Oxacillin (1 μg) discs were used to screen for penicillin non-susceptibility. Antibiotic discs (Thermo Fisher, Germany) were applied on the agar plates and incubated at 37 °C in 5% CO_2_ for 18–24 h. *S. pneumoniae* ATCC 49619 was included in each test batch as a control strain. Penicillin MIC test strips (Liofilchem) were used to measure the minimum inhibitory concentration (MIC) of oxacillin-resistant isolates. Isolates with MIC ≤ 0.06 μg/mL, 0.12 μg/mL, and ≥2 μg/mL were defined as penicillin-susceptible, penicillin-non-susceptible, and penicillin-resistant pneumococci, respectively. Isolates resistant to three or more classes of antibiotics were classified as multidrug resistant (MDR).

### 2.3. Serotyping and Molecular Characterization of Pneumococcal Strains

DNA was extracted from the pneumococcal isolates using the manufacturer’s instruction in the QIAamp DNA Mini Kit (Qiagen Diagnostics GmbH, Hilden, Germany). The extracted DNA was used as template DNA for all molecular tests. Primers and protocols described earlier [23] were used in a multiplex PCR (mPCR) to deduce pneumococcal serotypes. The serotypes were further confirmed using the Quellung reaction performed at the German National Reference Center for Streptococci, Aachen, Germany.

### 2.4. Virulence Gene Determination

Pneumococcal virulence genes including *lytA, pavB*, *pcpA*, *psrP*, the pilus islets (PI) *PI-1*, and *PI-2* were amplified with primers described earlier [11]. In brief, DNA templates from pneumococcal isolates were used in conventional PCR to amplify the virulence genes. Each reaction mixture contained 1 μL DNA extract, 1 μL of 25 mM MgCl_2_ (Roth), 1 μL of 5 mM dNTPs (Thermofisher), 2.5 μL of 10X Dream buffer (Thermofisher), 1 μL of the respective primers, 0.5 μL of DreamTaq DNA polymerase (Thermofisher), and nuclease-free water to make up 25 μL end volume. The peqSTAR 2X thermocycler (VWR) was used under these conditions: 4 min at 94 °C followed by 30 cycles composed of 30 sec at 94 °C, 30 s at 55 °C, 1–3 min at 72 °C (varies based on expected amplicon size), and 5 min at 72 °C. A 0.8% agarose gel was used to visualize the PCR fragments.

### 2.5. Multilocus Sequence Typing (MLST)

The internal fragments of the seven housekeeping genes *aroE*, *gdh*, *gki*, *recP*, *spi, xpt,* and *ddl* were amplified by PCR and sequenced. We used primers described in the PubMLST [24] database and alternative primers described by the CDC [23]. Amplifications for all genes were carried out with approximately 1 μL of 100 ng of DNA template, 1 μL of 25 mM MgCl_2_ (Roth), 1 μL of 5 mM dNTPs (Thermofisher), 5 μL of 10X Dream buffer (Thermofisher), 1 μL of the respective primers, and 0.5 μL of DreamTaq DNA polymerase (Thermofisher), and nuclease-free water in a 50 μL reaction mixture.

Thermocycling conditions were 4 min hold at 94 °C, followed by 30 cycles of 95 °C for 30 s, 55 °C for 30 s, 72 °C for 60 s, and a final extension at 72 °C for 5 min. The reaction mixture was separated by electrophoresis on a 0.8% agarose gel and visualized under UV illumination with a gel documentation system. The amplified genes were sequenced in both directions at Macrogen, Inc. (Amsterdam, The Netherlands). Sequence analysis was performed with DNADynamo (Blue Tractor Software Ltd, North Wales, UK). The consensus sequences were submitted to the PubMLST database and each gene sequence was assigned an existing or novel allele type number and sequence type (ST) numbers prescribed by the database. The relatedness of STs was analyzed using the goeBURST program [25]. Cluster analysis of related sequence types was grouped into clonal complexes (CCs) and illustrated using minimum spanning and neighbor-joining trees in the PHYLOViZ program.

### 2.6. Statistical Analysis

Data were analyzed using GraphPad Prism (GraphPad software^®^) version 5 (Dotmatics, San Diego, CA, USA) and Statistical Package for Social Sciences (SPSS) software^®^ (version 21) (IBM, New York, NY, USA). Categorical data were expressed as proportions and compared using the Chi-square test or Fisher’s exact test (two-tailed) where necessary.

### 2.7. Ethical Approval

The institutional review board of the University of Cape Coast granted ethical approval for this study (UCCIRB/EXT/2017/21). In addition to parental consent, all the children voluntarily participated in this study.

## 3. Results

**Figure 1:**
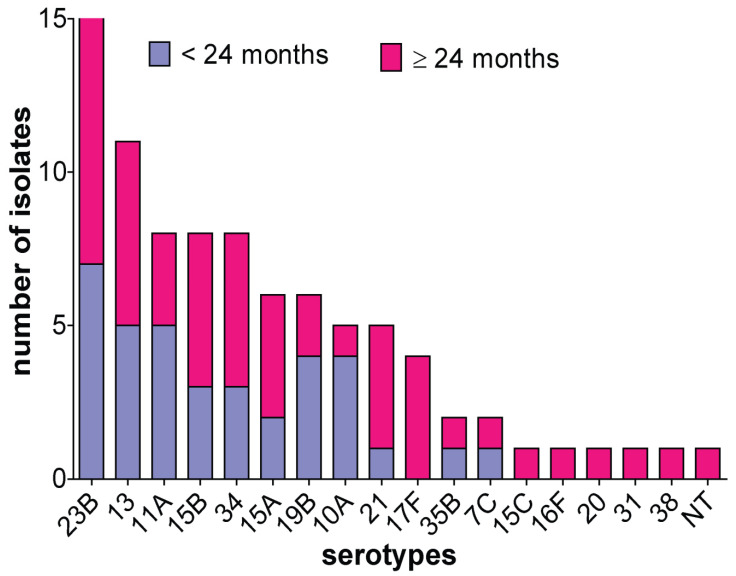
Non-PCV13 serotype distribution by age group. Age group ≤24 months includes children aged 6 months to 24 months and children 25 months to 59 months are included in the ≥24 months age group. NT, non-typeable.

A total of 93 NVT pneumococcal strains was isolated from the nasopharynx of 53 (57%) females and 40 (43%) male children under five years of age. More than half (*n* = 57, 61.3%) of the isolates were detected in children ≥24 months (Figure 1). A total of 17 different NVTs with one non-typeable strain was identified. The predominant serotypes were serotypes 23B (*n* = 22, 23.7%), 13 (*n* = 11, 11.8%), 11A, 15B and 34 (*n* = 8, 8.6%), and 15A and 19B (*n* = 6, 6.5%).

### 3.1. Antibiotics Susceptibility Patterns of NVT Isolates

Table 1 shows the antibiotic susceptibility patterns among the different serotypes in the study. All isolates were fully susceptible to vancomycin, levofloxacin, and linezolid. Furthermore, susceptibilities towards ceftriaxone, erythromycin, clindamycin, and chloramphenicol were >86% (Table 1). However, the data showed a marked non-susceptibility towards tetracycline 40 (43.0%) and cotrimoxazole 70 (75.4%). In contrast, penicillin non-susceptibility was relatively low, with 19 (20.4%) and 1 (1.1%) of the isolates being intermediate and resistant, respectively. Sixteen (17.2%) isolates were MDR, of which serotype 23B was the most prevalent. Furthermore, MDR to ≥4 different antibiotics was seen in five (5.4%) of the isolates and was limited to serotypes 23B, 35B, and 38, respectively.

### 3.2. Characterization of Pneumococcal Virulence Genes

The *lytA* and *pavB* genes were identified in all pneumococcal isolates; however, the pilus islets (*PI-1* and *PI-2*) were limited to serotypes 13, 11A, and 23B, respectively (Table 1). In contrast, *pcpA* and *psrP* genes were detected among the different serotypes in these proportions: 85 (91.4%) and 61 (65.6%), respectively.

### 3.3. Phylogeny of NVT Serotype 23B

**Figure 2:**
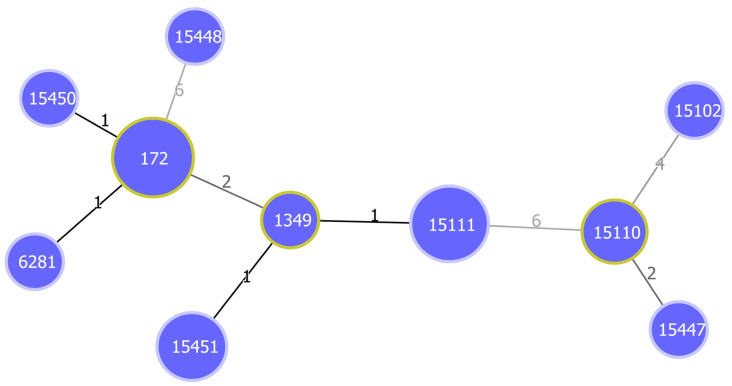
Minimum spanning tree (MST) showing the genetic relationship between STs obtained from MLST of serotype 23B strains. The tree was generated using the goeBurst algorithm in PHYLOViZ software. The diameters of nodes are proportional to the number of isolates. Founder STs have yellow color around their nodes. Branch labels correspond to the number of allelic variations between STs; branch lengths are not to scale.

**Figure 3:**
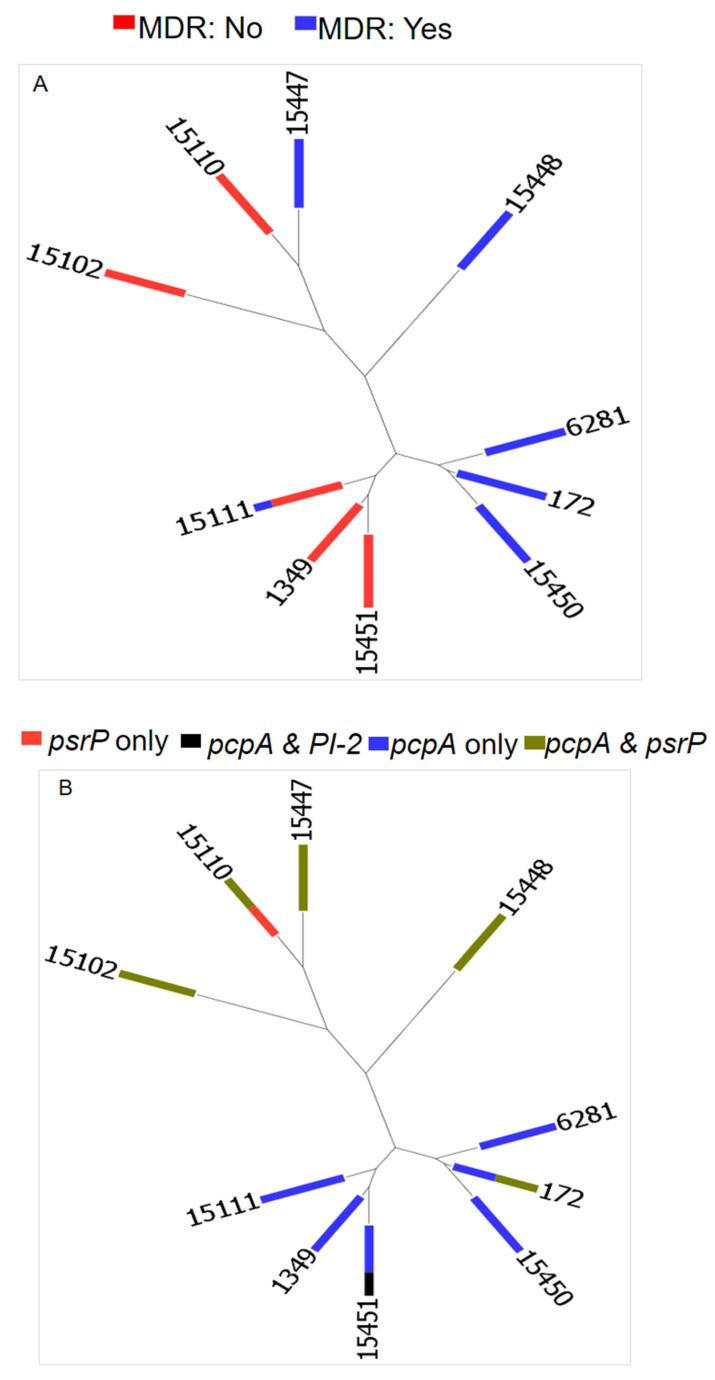
Relationship between STs, multidrug resistance and virulence genes. The neighbor-joining tree (NJT) was generated using PHYLOViZ. (**A**) Multidrug-resistance (MDR) relationship among the different STs. MDR was detected in both new and existing STs. (**B**) Distribution of virulence genes by STs. There was a fair distribution of all the virulence genes among the identified STs except the pilus islet 2, which was limited to only ST15451.

MLST was performed on the most prevalent NVTs (serotype 23B) to determine their genetic profiles. The MLST of the 22 serotype 23B isolates revealed three known STs and seven novel STs, containing 8 and 14 isolates, respectively (Table 2). The most common STs were ST172 (*n* = 6, 27.3%) and novel ST15111 (*n* = 5, 22.7%). The goeBurst algorithm (Figure 2) identified two notable clonal complexes, namely CC1 (ST172, ST6281, and ST15450) and CC2 (ST1349, ST15111, and ST15451). MDR was associated with both novel and already known STs (Figure 3A). Unlike the *pcpA*, *psrP*, *lytA*, and *pavB* virulence genes relating to the various STs, the *PI-1* gene was not associated with any ST and *PI-2* was present in ST15451 (Figure 3B).

**Figure 4:**
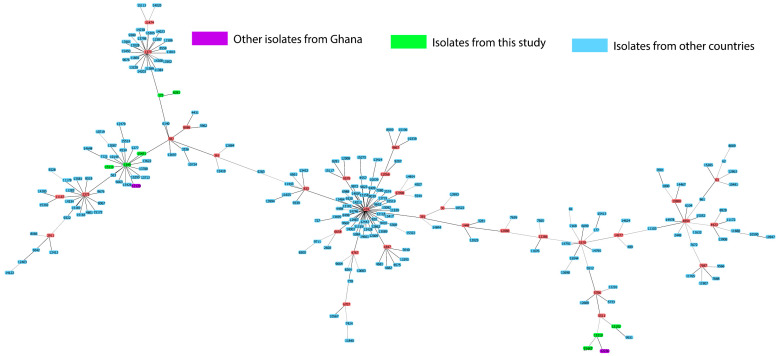
Genetic relatedness of serotype 23B isolates from this study compared with serotype 23B isolates from the PubMLST database. PubMLST database (accessed on 29 September 2020). Green color represents STs reported from this study, blue color, STs from other countries, purple color, STs of other serotype 23B strains from Ghana.

Figure 4 shows a comparison of the genetic relatedness of the serotype 23B isolates from this study to other serotype 23B isolates in the PubMLST database. Two isolates previously reported from Ghana were among the serotype 23B collection in the PuBMLST database. The novel ST15110 from this study varied at one locus from ST12236, found in a serotype 23B nasopharyngeal isolate reported from Ghana in 2011. In addition, the predominant ST172 varied at one locus from emerging clone ST1373l.

## 4. Discussion

Non-vaccine serotypes (NVTs) have emerged in countries where PCV vaccination has been introduced [15,19,20,26,27]. It is likely that NVTs were present prior to PCV introduction and have expanded to fill the niches vacated by VTs due to vaccine-induced selective pressure [27]. It is, therefore, important to continuously monitor and characterize these NVTs to fully understand their epidemiology and evolution.

Serotypes 11A, 13, 15A, 15B, 19B, and 23B are among the most common NVTs identified in this study. The emergence of replacement serotypes similar to those from this study has been reported in Ghana and several African countries that have introduced PCV in their routine immunization program [26,28,29]. Although some of these NVTs are considered to be of low potential to cause IPD, serotypes, such as 15B/C, 23B, 35B, and 38, are already associated with IPD in many countries [13,27,30]. Interestingly, the aforementioned serotypes have also been reported to be causal pathogens of pneumococcal disease among children under five years old in Ghana [27,31]. In this study, none of the isolates expressed serotype 12F, a non-PCV13 serotype that was implicated in a recent pneumococcal meningitis outbreak in Ghana and also found in IPD among children under five years [27,31].

Antibiotic-resistant pneumococcal isolates causing IPD declined after PCV introduction, partly due to the reduction in VTs, which were mostly associated with antibiotic resistance. However, the emergence of antibiotic-resistant NVTs identified in this study and similar to reports from previous studies emphasizes the need to maintain surveillance on NVTs [29,30]. There is a likelihood of these antibiotic-resistant NVTs progressing from carriage to cause IPD, which could eventually eliminate the full benefit of PCVs. Penicillin is the drug of choice for treating pneumococcal infections, yet increased penicillin resistance has been observed in populations where penicillin has been used extensively. Implementation of PCVs led to a drastic decline in penicillin resistance among pneumococcal isolates [11,20,32]. This decline in penicillin resistance among pneumococcal isolates causing IPD has been indicated as a positive effect of PCV vaccination [32]. In this study, we identified a relatively low resistance to penicillin, which contrasts with findings from other studies [27,33]. It is not surprising to observe penicillin resistance in NVTs, as this development could be due to the acquisition of resistance genes from other alpha-hemolytic streptococci present in the nasopharynx.

Although antibiotic resistance was low towards erythromycin, levofloxacin, and ceftriaxone, marked resistance to cotrimoxazole and tetracycline was noted. In contrast, a decline in cotrimoxazole resistance was reported among invasive pneumococcal isolates from children under five in Ghana [26,27]. However, our results are in accordance with previous post-PCV carriage studies conducted in Ghana [20]. It is noteworthy that similar antibiotic-resistance patterns were observed in the pre-vaccination era [34,35]. Therefore, the marked antibiotic resistance seen among the NVTs could be attributed to the acquisition of resistance genes and the uncontrolled use and abuse of antibiotics by the general public. Furthermore, cotrimoxazole continues to be administered in the treatment of common respiratory infections among children in Ghana and also as prophylaxis in HIV treatment [27,36]. Our results show that serotypes 15A, 23B, and 35B were multidrug resistant and this finding is in agreement with previous studies [33]. Other studies [27,33] that identified similar trends attributed the MDR seen in these serotypes to their genetic complexities. In this regard, these serotypes could become dominant strains with limited treatment options in the future.

In this study, we showed that all the NVT isolates possessed *lytA* and *pavB* genes, with >65% testing positive for *pcpA* and *psrP* genes. The proteins encoded by these genes are, hence, highly conserved in our NVT strain collection, similar to what was detected among VTs [11]. This finding suggests that these proteins cover multiple serotypes and could offer protection when included in protein-based vaccines. In contrast, the detection of pilus islets was very low and limited to serotypes 11A, 13, and 23B. Although previous studies identified pilus islets predominantly in VTs [37], recent studies [38] have also demonstrated the emergence of piliation among NVTs. The presence of pilus islets has been linked to antibiotic resistance and it is, therefore, not surprising that piliated serotypes from this study show antibiotic resistance. It is, therefore, important to monitor piliation in NVTs as they could expand and emerge in IPD and become associated with treatment difficulties as well.

Previous post-PCV data from Ghana [20] identified serotype 23B as a dominant strain, a finding which concurs with our findings. However, data on the molecular characteristics of this emerging serotype are lacking in Ghana. In this study, we, therefore, explored the genetic background of 22 serotype 23B isolates. The most frequently occurring ST was ST172, which is associated with serotypes 23F and 23B, with the majority of the isolates expressing penicillin non-susceptibility [26,27]. ST172 seems to be a global clone as it has been reported by several African countries, the USA, Australia, Europe, Asia, and the Middle East [24]. It is, however, interesting to note that ST172 is a single locus variant (SLV) of ST338 (Colombia23F-26), a PMEN clone that has been linked to the global spread of penicillin resistance [39]. Hence, it is not surprising to see that the study isolates of ST172 were all penicillin resistant. Because vaccines induce a serotype-specific immune response, some serotypes switch their capsules in order to evade the immune system. It is, therefore, possible that serotype 23B strains from this study associated with ST172 could have evolved from serotype 23F. A study in the USA reported that serotype 23A belonging to ST172 shared the same genetic background as serotype 23F strains, suggesting an expansion of the clone as well as a capsular switch [40].

The other known STs have been reported solely by South Africa (ST6281), whereas ST1349 has been reported primarily from Europe and the USA [24], with >90% of the isolates presenting as serotype 23B and being penicillin non-susceptible. This observation suggests the dissemination of these clones beyond their previously identified geographical areas. On the one hand, ST6281 is associated with serotype 23F [24]. However, in our study, ST6281 is of serotype 23B, demonstrating yet another natural capsule switch event. This event could occur as a result of the high homology between serotypes 23F and 23B [41]. Other studies have reported such events between serotypes 6A and 6C [42]. On the other hand, ST1349 is a double locus variant (DLV) of ST338 (Colombia^23F^-26), with a serotype 23F genetic background. The similarities in the genetic backgrounds observed between serotypes 23F and 23B suggest a possibility of clonal expansion of the previously existing clones, which could have been present in the pre-vaccination era. In addition, findings from a recent study suggest that closely related serotypes, such as (6B/6C, 15B/15C, 35F/35D), could switch their capsules and yet maintain the same ST over a period [43].

Despite the possible change in the capsule, the genetic background remains highly identical to that of serotype 23F of ST338 (Colombia^23F^-26). Out of the seven novel STs, three were SLVs of known STs, indicating a form of clonal expansion of their founding ancestor. In addition, two new STs were DLVs of the PMEN clone ST338 (Colombia^23F^-26), which supports the evidence of penicillin resistance seen among these STs. It is interesting to note that some of the novel STs already expressed MDR. Therefore, the expansion of these clones in the future could be problematic as treatment options may be limited.

The only pilus possessing serotype 23B strain belonged to the novel ST15451, showing resistance to penicillin but not being multidrug resistant. Similarly, the presence of PI-2 among pneumococcal strains in Canada did not influence their propensity to drug resistance [44]. The other virulence genes (*pcpA* and *psrP)* were evenly distributed among all the STs, except *PI-1*, which was not detected among any of the STs. This suggests that including PcpA and PsrP in protein-based vaccines could be beneficial in the long term to fight emerging NVTs.

We compared the STs of our serotype 23B with STs of all serotype 23B present in the PubMLST database. Interestingly, the STs identified in this study did not cluster around the predominant clone ST439, which has been reported to circulate in Europe. ST439 is the major clone associated with serotype 23B strains circulating in Germany [13]. However, the predominant clone ST172 found in this study appeared as an SLV of ST1373, which is a subgroup founder that has been reported mainly by the USA. The two other subgroup founders associated with STs from this study were ST1349 and ST5511. These STs (ST1373, ST1349, and ST5511) are quite distant from the dominant clone ST439.

## 5. Conclusions

To conclude, this study describes the serotype distribution of NVTs observed in pneumococcal carriage isolates from vaccinated children less than five years of age in Cape Coast, Ghana. Despite the recorded low antibiotic resistance to penicillin, marked resistance towards cotrimoxazole and tetracycline was observed. The studied virulence genes were present among all the NVTs, except for the pilus islets, which were limited to specific serotypes. ST172, an SLV of PMEN clone ST338 (Colombia^23F^-26), was the predominant clone among the serotype 23B isolates. This underscores the need for continuous monitoring of NVTs, their molecular characteristics, and antibiotic susceptibility patterns in carriage and IPD.

## Figures and Tables

**Table 1:** Distribution of virulence genes and antibiotic non-susceptibility patterns of non-PCV13 serotypes in Cape Coast, Ghana.

Pneumococcal Isolates		Antibiotic Non-Susceptibility								Virulence Genes			
Serotypes	Number	CRO	ERY	CLI	TET	CHL	COT	PEN	MDR	*pcpA*	*psrP*	*PI-1*	*PI-2*
23B	22	0	4.5	4.5	50	12.5	86.4	72.7	45.5	95.5	36.4	0	4.5
13	11	0	0	0	72.7	27.3	72.7	0	0	100	90.9	9.1	0
11A	8	0	0	0	12.5	0	87.5	12.5	0	100	87.5	12.5	0
15B	8	0	0	0	25	12.5	100	0	0	100	75	0	0
34	8	0	0	0	12.5	12.5	37.5	0	0	75	87.5	0	0
15A	6	16.7	0	0	83.3	16.7	100	16.7	50	83.3	66.7	0	0
19B	6	0	0	0	83.3	0	66.6	0	0	100	83.3	0	0
10A	5	20	0	0	0	0	60	0	0	100	20	0	0
21	5	0	0	0	100	0	100	0	0	80	100	0	0
17F	4	0	0	0	25	0	25	0	0	100	0	0	0
35B	2	0	100	100	100	0	0	50	100	100	100	0	0
7C	2	0	0	0	100	0	0	0	0	0	100	0	0
15C	1	0	0	0	0	0	100	0	0	100	0	0	0
16F	1	100	0	0	100	0	0	0	0	100	100	0	0
20	1	0	0	0	0	0	0	0	0	100	100	0	0
31	1	0	0	0	0	0	100	0	0	100	100	0	0
38	1	0	100	0	100	0	100	100	100	100	100	0	0
NT	1	0	0	0	100	0	100	0	0	0	0	0	0
Total	93 (100)	3 (3.2)	4 (4.3)	3 (3.2)	45 (48.4)	7 (7.5)	70 (74.3)	20 (21.5)	16 (17.2)	85 (91.4)	61 (65.6)	2 (2.2)	1 (1.1)

Percentage of isolates (%), presence of virulence genes is expressed in percentages. CRO, ceftriaxone; ERY, erythromycin; CLI, clindamycin; TET, tetracycline; CHL, chloramphenicol; COT, cotrimoxazole; PEN, Penicillin. Antibiotic non-susceptibility (includes intermediate and full resistance isolates). All the isolates were susceptible to vancomycin, levofloxacin, and linezolid. Multidrug resistant (MDR), ≥3 classes of antibiotics. *pcpA*, pneumococcal choline-binding protein A; *psrP*, Pneumococcal serine-rich repeat protein; *PI-1*, Pilus islet 1; *PI-2*, Pilus islet 2.

**Table 2:** MLST of serotype 23B pneumococcal isolates.

Isolate ID	*aroE*	*gdh*	*gki*	*recP*	*spi*	*xpt*	*ddl*	ST
S119, S110, S158, H174, S258, S357	7	13	8	6	25	6	8	172
S114	7	13	8	6	25	246	8	6281
S311	7	19	8	6	25	6	8	**15450**
S1	12	5	4	10	15	155	9	**15102**
S152, S239	12	5	4	18	474	4	31	**15110**
S200, S207, S214, S238, S361	12	13	8	6	3	6	8	**15111**
S35	18	13	8	6	3	6	8	**1349**
H58, S266, S306	18	13	8	6	3	21	8	**15451**
S156	2	5	36	18	474	4	31	**15447**
S237	1	43	41	18	13	37	8	**15448**

Bold numbers: novel STs.

## Data Availability

Data set for this study is available upon request from the authors.
